# Transformer based on channel-spatial attention for accurate classification of scenes in remote sensing image

**DOI:** 10.1038/s41598-022-19831-z

**Published:** 2022-09-14

**Authors:** Jingxia Guo, Nan Jia, Jinniu Bai

**Affiliations:** Baotou Medical College, Baotou, 014040 Inner Mongolia China

**Keywords:** Engineering, Mathematics and computing

## Abstract

Recently, the scenes in large high-resolution remote sensing (HRRS) datasets have been classified using convolutional neural network (CNN)-based methods. Such methods are well-suited for spatial feature extraction and can classify images with relatively high accuracy. However, CNNs do not adequately learn the long-distance dependencies between images and features in image processing, despite this being necessary for HRRS image processing as the semantic content of the scenes in these images is closely related to their spatial relationship. CNNs also have limitations in solving problems related to large intra-class differences and high inter-class similarity. To overcome these challenges, in this study we combine the channel-spatial attention (CSA) mechanism with the Vision Transformer method to propose an effective HRRS image scene classification framework using Channel-Spatial Attention Transformers (CSAT). The proposed model extracts the channel and spatial features of HRRS images using CSA and the Multi-head Self-Attention (MSA) mechanism in the transformer module. First, the HRRS image is mapped into a series of multiple planar 2D patch vectors after passing to the CSA. Second, the ordered vector is obtained via the linear transformation of each vector, and the position and learnable embedding vectors are added to the sequence vector to capture the inter-feature dependencies at a distance from the generated image. Next, we use MSA to extract image features and the residual network structure to complete the encoder construction to solve the gradient disappearance problem and avoid overfitting. Finally, a multi-layer perceptron is used to classify the scenes in the HRRS images. The CSAT network is evaluated using three public remote sensing scene image datasets: UC-Merced, AID, and NWPU-RESISC45. The experimental results show that the proposed CSAT network outperforms a selection of state-of-the-art methods in terms of scene classification.

## Introduction

The rapid development of remote sensing technology and the continuous increase in the number of satellites has provided a wealth of data sources for ground surveys^1–3^, as well as a solid foundation for the interpretation of complex and high-resolution remote sensing images. Remote sensing image classification has been extensively researched, laying the foundation for the effective analysis of practical applications such as urban planning^4^, geospatial object detection^5,6^, vegetation mapping^7^, and environmental monitoring^8^. Remote sensing image classification is generally divided into three categories: pixel-, object-, and scene-level^9^. With the continuous improvement in remote sensing image resolution, and an increasing number of images containing different target categories, pixel- and object- classification methods can no longer meet the requirements for the accurate classification of complex remote sensing images. Accordingly, researchers have recently adopted a method to automatically classify images with a specific semantic label based on the content of the entire image (i.e., remote sensing image scene classification) to extract high-level semantic information from remote sensing images^10^ and provide auxiliary references to understand what appears in the images. However, scene classification capable of accounting for image semantics is still a challenging task due to the complex spatial distribution of objects in scenes and the diverse types of land cover^11^.

The primary goal of high-resolution remote sensing (HRRS) image scene classification is to correctly classify a given remote sensing image according to its content (e.g., commercial, industrial, or residential areas)^12^. Classification performance largely depends on features that accurately represent the scene in the image, and thus the extraction of features that describe an image more accurately has become a primary research focus. Recently, convolutional neural networks (CNNs) have been widely utilized in scene classification because they are capable of extracting high-level semantic feature representations for scene classification^13–15^. However, the spatial relationships between features in HRRS images are complex, and there is a large amount of redundant information. Consequently, it is difficult to directly extract the features that reflect the key information of the image content. The human visual attention mechanism involves obtaining detailed information of the target region by scanning the global area. Similarly, the attention mechanism in CNNs simulates the way humans understand and perceive images by assigning different weights to global features, highlighting key local features, and suppressing invalid features. For example, Park et al.^16^ proposed a simple and effective bottleneck attention module (BAM) that can be integrated into any CNNs architecture. It focuses on high-level semantic features by allocating feature weights to input images through an effective combination of spatial- and channel-independent paths. Woo et al.^17^ proposed a convolutional block attention module (CBAM), which is a lightweight general attention module. CBAM infers the attention map along the spatial and channel dimensions and then assigns weights to features, which can be seamlessly integrated into CNN architectures. In addition, Yu et al.^18^ adopted an improved channel attention mechanism to enhance features at different levels. Furthermore, Tong et al.^19^ introduced an attentional mechanism in the channel dimension that adaptively enhanced the weights of important feature channels and inhibited secondary feature channels. Remote sensing images not only contain rich channel information but also spatial information. To fully extract their features, Ma et al.^20^ proposed an adaptive multi-scale spatial attention module (AMSA) and an adaptive multi-scale channel attention module (AMCA) based on image characteristics by adopting adaptive expansion rate selection strategy (ADR-SS), so as to increase the diversity of extracted features. Zhu et al.^21^ designed an adaptive spatial attention module (ASA-Module) and an adaptive channel attention module (ACA-Module) to strengthen spatial features from both larger- with smaller-sized targets and spectral features among channels. Zhu et al.^22^ designed a spatial attention module (SA-module) and a channel attention module (CA-module), Ma et al.^23^ designed a local spatial attention module (LSA-module) and a global channel attention module (GCA-module), which not only highlight the advantages of spatial resolution and channel features but also reduced the difference between features through the interaction between the two modules. Li et al.^24^ adopted an enhanced attention mechanism to prompt the beneficial information in both spatial and channel dimensions to push the model to capture discriminative regions as much as possible. Guo et al.^25^ proposed a global–local attention network (GLANet), which assigns different weights to different channels through global branch learning, and local branch learning to improve relevant spatial attention regions and weaken background regions. These previous studies demonstrate that channel and spatial attention mechanisms play a certain role in enhancing the main features, and decisions are made on this basis according to the needs of the model. Based on this property, our method first adopts the channel-spatial attention mechanism to focus on the key information in the image and form an attention map, which is used as the input to the encoder.

Although CNNs excel in spatial feature extraction and can achieve relatively high classification accuracy, there are some limitations. First, the receptive field of CNNs is limited by the size of the convolution kernel, which introduces difficulties in capturing global information. Second, CNNs are not suitable for mining long-range dependencies inside image scenes. The potential spatial topological relationship can be readily ignored, and CNNs still have certain restrictions in processing large and small intraclass differences. Third, increasing the number CNN layers can extract more features and increasing the size of the convolution kernel can obtain a larger receptive field; however, this will greatly increase the complexity of the model and lead to the gradient disappearance problem.

Recently, transformers^26^ have been applied in various vision tasks due to their excellent ability to capture long-range dependencies and sequence-based image modeling. For example, the Vision Transformer (ViT) demonstrates that the standard transformer architecture can achieve state-of-the-art performance in image classification^27^. On this basis, Bazi et al.^28^ applied the standard transformer structure to HRRS image classification and achieved better classification accuracy than through other advanced classification models. In addition, Deng et al.^29^ proposed CTNet, which mines the semantic features in HRRS scene images through ViT and extracts local structural features in HRRS scene images via a CNN. Finally, these two features are combined to classify HRRS scenes. However, when a transformer processes an image, the image must be divided into patches, which limits the ability of the model to learn the overall image characteristics. Therefore, Li et al.^30^ proposed the remote sensing transformer (TRS), which uses self-attention integrated into a residual neural network (ResNet), Multi-Head Self-Attention (MHSA) layers instead of spatial convolutions, and concatenates multiple pure transformer module encoders to improve the attention-dependent representation learning performance. Ma et al.^31^ proposed a homo- heterogenous transformer learning (HHTL) framework for HRRS scene classification according to the characteristics of the transformer to divide the image into multiple patches.

To address the aforementioned problems, we designed a new HRRS image scene classification framework based on a channel-spatial attention transformer (CSAT). First, the HRRS scene image is divided into patches, and local fine-grained features are extracted via the BAM attention mechanism to enhance the spatial and channel features, thereby reducing redundant information in the image and improving the ability to obtain local information. Then, the image is transformed into a sequence via linear transformation, during which embedding positions are added to the patches to preserve positional information. Finally, the transformer module was used as an encoder to extract the image features, which can effectively learn global and local context information. The experimental results for three public datasets used for HRRS image scene classification demonstrate the effectiveness of the proposed algorithm.

In this paper, we choose transformer instead of CNNs for feature extraction for the following reasons. First, transformer is a new encoder-decoder architecture that relies on an attention mechanism to characterize the global dependencies between its input and output^32^, overcoming the convolutional inductive bias of CNNs on the overall input data insufficient grasp. Furthermore, CNNs cannot effectively extract long-distance dependent features between global data^33^. Second, the feature maps generated by the self-attention mechanism in transformer do not have the same spatial constraints as convolution calculations, can handle a larger receptive field than conventional convolutions^34,35^, and can effectively obtain global information. It is beneficial to explore the global contextual knowledge hidden in HRRS scenarios. To obtain global information, CNN needs to expand the receptive field, use larger convolution kernels, and stack deeper convolution layers. However, as the number of layers increases, the amount of information will be exhausted, and dimensional disasters may occur. Third, the self-attention mechanism layer in transformer will have a large amount of calculation for high-resolution input data, while being more suitable for processing data scenarios with smaller spatial dimensions. Therefore, it is possible to process only small feature maps instead of the entire feature map space, which will inevitably result in a relatively small receptive field, which, however, is larger than the receptive field of the convolution kernel of the convolution operation^36^. This processing method is more suitable for mining more useful information from complex HRRS scene images.

The main contributions of this study can be summarized as follows.

- A modified Transformer network model titled CSAT is designed to complete the HRRS scene classification task. This method uses the channel-spatial attention mechanism and self-attention mechanisms to extract feature information and avoid the loss of feature information.
- The channel-spatial attention mechanism helps improve the network's ability to obtain local information. It focuses on fine-grained features in patches according to two independent paths (channel and space), which mitigates the effects of small differences between classes and large differences within classes.
- HRRS produces various scenario categories and rich scenario information. The core process of the network is a multi-head self-attention encoder block, which successfully handles the long-range dependence of the spatial information in HRRS images.
- The CSAT network introduces the transformer structure as an encoder to avoid the dimension disaster caused by too many layers and enhance the global modeling ability of the network.
- Our proposed CSAT network is interpretable and offers an enhanced capability in extracting HRRS image features and generalizations.
- We propose a CSAT learning scheme that combines the contributions mentioned above. Experiments were conducted on three different scene classification datasets, and the results demonstrate that the proposed method outperforms state-of-the-art methods in terms of scene classification.

## Methods

**Figure 1:**
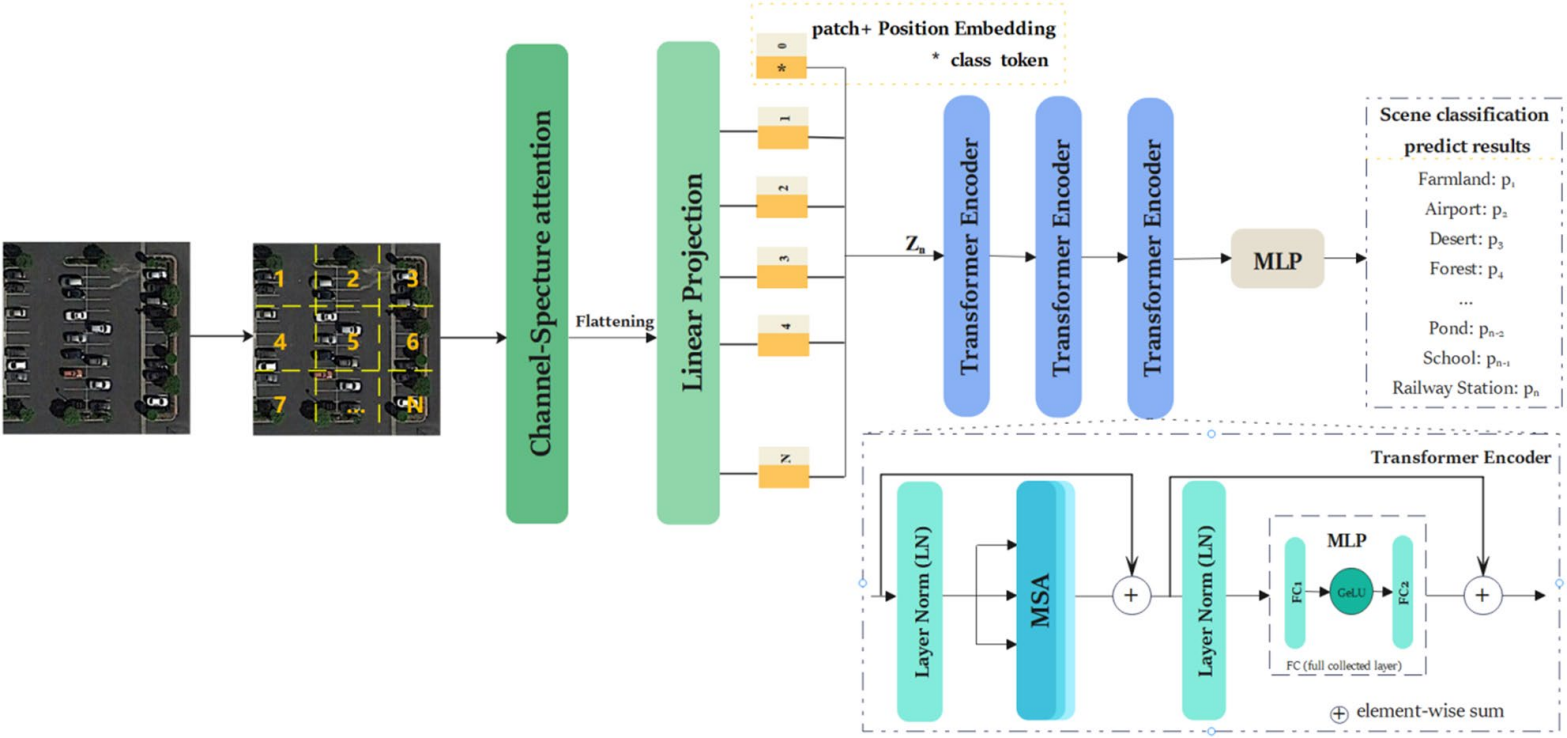
Structure of the channel-spatial attention transformer (CSAT) based on the transformer and channel-spatial attention module.

The overall architecture of the CSAT is shown in Fig. 1, where the image input is sliced into evenly sized patches and sequential patches are fed into the CSA module to infer the attention patch (a detailed explanation is presented in the following section). The attention patches are then transformed into a vector of patch embeddings via flattening and linear projection. The embedding position is added to this projection and the category identity is sent as input to the transformer encoder along with the patch embedding vector. After a multi-layer perceptron (MLP) classifier is used for classification, the probability values P_1_, P_2_, …, P_n_ are obtained for each category.

### Long-range-dependent feature extraction of high-resolution remote sensing images

#### Channel-spatial attention mechanism for HRRS feature extraction

Attention mechanisms play an important role in the human visual system by enabling the neural network to selectively focus on salient parts, removing redundant information, and efficiently extracting important features from images such as those obtained from HRRS.

**Figure 2:**
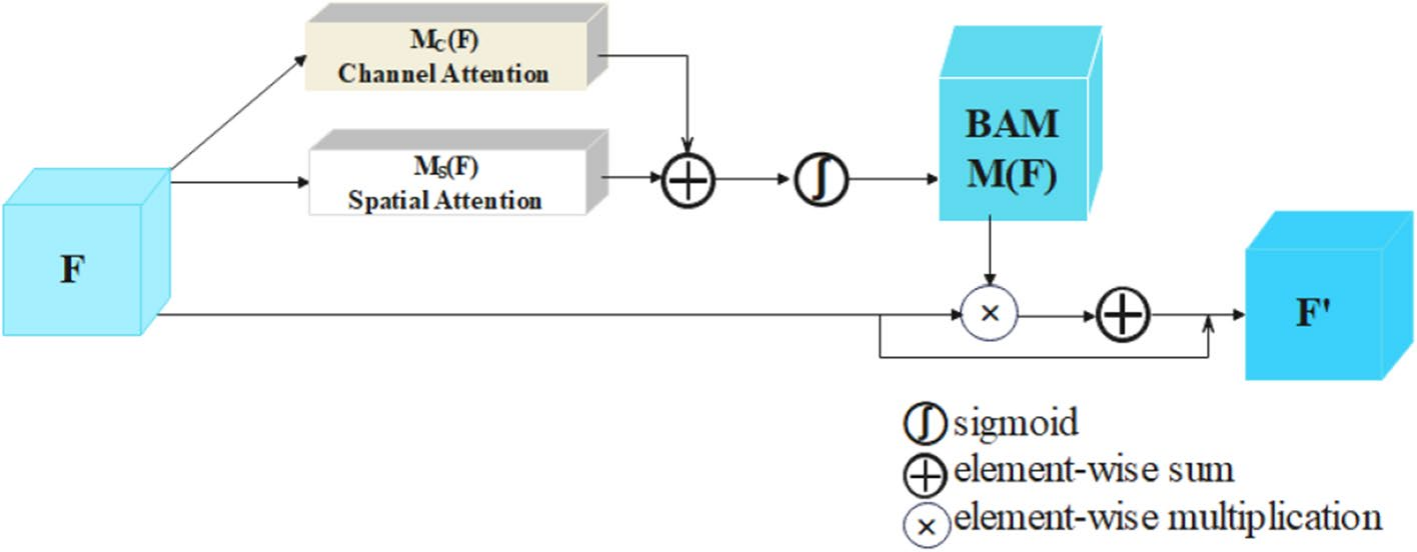
Channel-spatial attention mechanism (CSAM).

Attention mechanisms are widely used in computer vision tasks. For HRRS images with complex spatial information, we introduced a spatial-channel attention mechanism called the bottleneck attention module (BAM)^16^ (Fig. 2). This mechanism consists of two independent attention modules, the channel attention module (CAM) and the spatial attention module (SAM). To emphasize or suppress the information in a remote sensing image, CAM uses the inter-channel relationship and SAM uses the features of different spatial locations, respectively. After acquiring the attention maps separately, the "residual structure" proposed by ResNet^37^ was used to generate the refined feature map ${\text{F}}^{^{\prime}}$. The process by which the CAM and SAM image feature extraction is performed is described in detail below.

**Figure 3:**
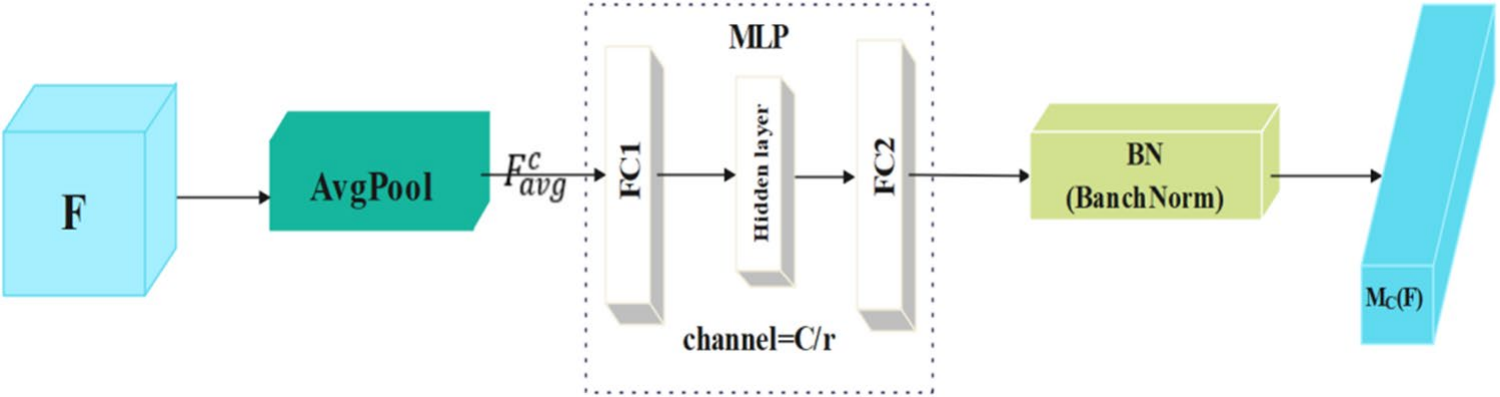
Channel attention module (CAM).

**Figure 4:**
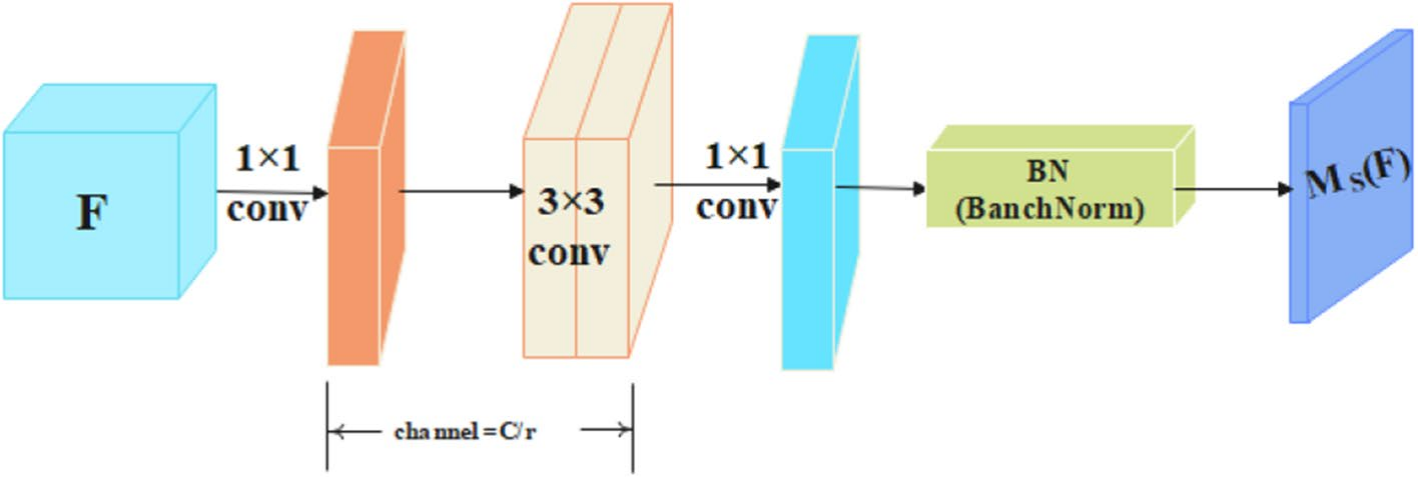
Spatial attention module (SAM).

1. The CAM process (shown in Fig. 3) is described as follows.
  Because each channel contains a specific feature response, CAM exploits the relationships between channels to aggregate the feature maps in each channel and generates the attention map ${\mathrm{M}}_{\mathrm{c}}(\mathrm{F})$. First, the feature map $\mathrm{F}\in {\mathbb{R}}^{C\times H\times W}$ is passed through the global AvgPool to obtain ${F}_{avg}^{c}$ for 1 × 1 × *C* channels, and then it enters the MLP with a hidden layer. To reduce the number of parameters, the activation size of the hidden layer was set as ${\mathbb{R}}$ ^C/*r*×1×1^, where *r* is the compression rate. Second, to adjust the scale of the CAM output, ${F}_{avg}^{c}$ is obtained as ${M}_{c}\left(F\right)\in {\mathbb{R}}^{C\times 1\times 1}$ after entering the batch normalization (BN) layer of the MLP. The CAM was computed as:
  1 $$\begin{array}{c}{\mathrm{M}}_{\mathrm{c}} (F)=BN\left(MLP\left(\mathrm{AvgPool}\left(\mathrm{F}\right)\right)\right) ,\\ \quad \quad =BN\left({\mathrm{W}}_{1}\left({\mathrm{W}}_{0}\mathrm{AvgPool}\left(\mathrm{F}\right)+{\mathrm{b}}_{0}\right)+{\mathrm{b}}_{1}\right),\end{array}$$
  where $${W}_{0}\in {\mathrm{R}}^{C/r\times C},{\mathrm{b}}_{0}\in {\mathrm{R}}^{C/r},{W}_{1}\in {\mathrm{R}}^{C\times C/r},{\mathrm{and b}}_{1}\in {\mathrm{R}}^{C}$$.
2. The SAM process (shown in Fig. 4) is described as follows.

The SAM generates spatial attention maps ${\text{M}}_{\text{s}}\left({\text{F}}\right)$, which are used to emphasize or suppress features in different spatial locations. First, the input feature map $\mathrm{F}\in {\mathbb{R}}^{C\times H\times W}$ is subject to a 1 × 1 convolution operation, and then the F dimension is reduced to ${\mathbb{R}}^{C/r\times H\times W}$ to integrate and compress F across the channel dimensions, where *r* = 16 is consistent with the setting found in the literature^16^. Second, we adopted two 3 × 3 convolutions to extract useful information from the context, and then adjusted the spatial attention map dimension to ${\mathbb{R}}^{1\times H\times W}$ through a 1 × 1 convolution. Finally, to adjust the scale of the SAM output, ${\text{M}}_{\text{s}}\text{(F)}\in {\mathbb{R}}^{1\times H\times W}$ was obtained after the BN layer. The SAM is described as:

2 $${\mathrm{M}}_{\mathrm{s}} \left(\mathrm{F}\right)=BN\left({f}_{3}^{1\times 1}\left({f}_{2}^{3\times 3}\left({f}_{1}^{3\times 3}\left({f}_{0}^{1\times 1}\left(\mathbf{F}\right)\right)\right)\right)\right),$$

where ${\mathrm{f}}^{i\times i}$ represents an $i\times i$ convolution operation with $i$ =1, 3.

### Transformer encoder for HRRS classification

The transformer^18^ mainly relies on the self-attention mechanism to construct a global dependency model between the input and output. Additionally, a standard transformer module usually includes multi-head self-attention (MSA), MLP, and layer norm (LN)^38^. Our transformer encoder was composed of three standard transformer modules, with the MSA module as the core part of the transformer module. In contrast to CNNs, which use convolutional and pooling layers to obtain feature information, the data used to extract features predominantly contains local information, and the ability to capture global information is poor. These conditions are not conducive to obtaining global spatial information in HRRS images or semantic information between images. The transformer extracts global features based on the attention mechanism and learns long-range dependencies, which helps encode patches according to global contextual information and captures the information between ordered patches, thus improving the performance of global feature extraction from HRRS images.

Specifically, the patches are first flattened, then positional embedding is added to the patch embedding vector Z_n_ to maintain the spatial location information between the input patches. Then, the learnable embedding vector for category classification is input to the transformer encoder along with Z_n_. At this point, a set of sequential patches are input to the transformer encoder, as shown in Fig. 1. Two important components of the transformer module are MSA and the MLP, which are computed via Eqs. (3) and (4), respectively.

3 $${z}_{l}^{\mathrm{^{\prime}}}=\mathrm{MSA} \left(\mathrm{LN}\left({z}_{l-1}\right)\right)+{z}_{l-1},l=\mathrm{1,2}\dots L,$$

4 $${z}_{l}=\mathrm{MLP} \left(\mathrm{LN}\left({z}_{l}^{\mathrm{^{\prime}}}\right)\right)+{z}_{l}^{\mathrm{^{\prime}}},l=\mathrm{1,2}\dots$$

The latter consists of two fully connected (FC) layers and an activation function, the Gaussian Error Linear Unit (GeLU). Residual connections are used in both components in the transformer module, and each component is preceded by an LN.

Among the above components, the MSA block is the central part of the transformer module, which can learn rich semantic features from patches of sequence of size *n*, capture internal data correlations, and establish dependencies among different features.

Specifically, each element in the input sequence Z is multiplied by three learnable weight matrices W_QKV_, which are composed of three values: *Q*, *K* and *V* (“query”, “key”, and “value” of dimensions D_q_, D_k_ and D_v_, respectively). These values can be calculated as:

5 $$\left[Q,K,V\right]=z{W}_{QKV}, { W}_{QKV}\in {\mathbb{R}}^{d\times 3{D}_{k}}.$$

To determine the correlation between elements in the sequence, the dot product between the Q-vector of that element and the K-vectors of the other elements was calculated, and the result determined the relative importance of the patches in the sequence. The softmax function was used to calculate the weights of V. Subsequently, the value of each patch embedding vector was multiplied by the output of the softmax function to obtain the patches with higher attention, which were calculated according to

6 $$\mathrm{Attention}\left(Q,K,V\right)=\mathrm{softmax}\left(\frac{Q{K}^{T}}{\sqrt{{D}_{k}}}\right)V.$$

MSA uses the previous operation multiple times to perform multiple dot-product attention calculations for *Q*, *K*, and *V* (i.e., *h* times), and then connects the results of these attentions via Eq. (7), which is the MSA process.

7 $$\mathrm{MultiHead}\left(Q,K,V\right)=\mathrm{concat}\left({\text{ Head}}_{1},\cdots ,{\text{ Head}}_{h}\right){W}^{O}.$$

Each result of these parallel attention calculations is called a head, which are defined as:

8 $${\text{Head}}_{i}=\text{ Attention }\left(Q{W}_{i}^{Q},K{W}_{i}^{K},V{W}_{i}^{V}\right),$$

where $${W}_{i}^{Q}\in {\mathbb{R}}^{\mathrm{d}\times {D}_{q}},{W}_{i}^{K}\in {\mathbb{R}}^{\mathrm{d}\times {D}_{k}},{W}_{i}^{V}\in {\mathbb{R}}^{\mathrm{d}\times {D}_{v}}, and {W}^{O}\in {\mathbb{R}}^{h\times {D}_{v}\times \mathrm{d}}$$.

The weights extracted via MSA are sent to the MLP layer, where an LN is performed before entering the MLP layer^38^. The LN serves not only to reduce the training time, but also to mitigate the effects of gradient disappearance and explosion. The MLP layer is composed of two fully connected (FC) layers, and the nonlinearity between the layers is called the activation of the GELU^39^, which is calculated as:

9 $$\mathrm{GELU}=\mathrm{X\Phi }\left(\mathrm{x}\right)=\mathrm{X}\cdot \frac{1}{2}\left[1+\mathrm{erf}\left(\frac{\mathrm{X}}{\sqrt{2}}\right)\right],$$

where $\Phi \left(\mathrm{x}\right)$ is the standard Gaussian cumulative distribution function and $\mathrm{erf}\left(x\right)={\int }_{0}^{x}{e}^{-{t}^{2}}dt$.

### Details of the patch size

An initial HRRS image of size (*w,h,o*) was mapped onto a set of images of size (*l,l,o*), which were first divided into patches of size (*p,p,o*) with sample data of size (*m,p,p,o*), where *p* is the height and width of the sample and $m=(l\times l)/(p\times p)$ is the number of samples. Second, the patches are passed through the CSAM module to redistribute the weights of the channel and spatial information. Because the CSAM does not change the shape of the input feature map, the shape of the output sample data remained (*m,p,p,o*). Finally, the output of *m* samples were flattened, and the 2D patch of size (*p,p,o*) with a sequence was entered into the transformer encoder after adding position encoding and category identification. The size of *p* was the same as the size of the patch designed by Dosovitskiy^26^. The purpose of this design strategy was to verify that the CSAM module can focus on the local features of the ordered patches before entering the transformer encoder, thereby improving the performance of the model, as described later.

## Experimental results and analysis

### Dataset description

In our experiments, three public remote-sensing datasets were used: the University of California Merced Land Use Dataset (hereafter “UCM”), the Aerial Image Dataset (hereafter “AID”), and the Northwestern Polytechnical University NWPU-RESISC45 Dataset (hereafter “NWPU”). The characteristics of each dataset are listed in Table 1. AID and NWPU are large-scale datasets.

**Table 1:** Characteristics of the dataset.

Dataset	Number of classes	Number of images/class	Image size	Total	Publishing organization	Ref
UCM	21	100	256 × 256	2100	United States Geological Survey	^40^
AID	30	220–420	600 × 600	10,000	Wuhan University	^41^
NWPU	45	700	256 × 256	31,500	Northwestern Polytechnical University	^42^

### Training details

We conducted all the experiments on a Dell Precision station with the following technical specification: an Intel(R) Xeon(R) Silver 4216@2.10 GHz central processing unit (CPU) with 64 GB of RAM and an NVIDIA RTX A4000 graphical processing unit (GPU) with a 16-GB memory. The code used for the experiments was implemented using PyTorch, an open-source deep neural network library written in Python. We used the Adam e optimizer with an initial learning rate of 0.001 and a weight decay of 0.00001. All experiments were trained using 300 epochs. We set the size of the UCM, NWPU, and AID datasets to 224 × 224 and the batch size to 64. We used 12 transformer blocks as encoders, the number of MSA headers in the transformer block was 12, the patch size was 16, and the ImageNet1K pre-trained parameters were used in the encoders.

### Comparison with the state-of-the-art methods

The main purpose of this study is to demonstrate that channel and spatial attention mechanisms optimize the transformer, which can improve the network performance. We used the overall accuracy as the evaluation criterion for this model, and all the experiments results used in the comparison were obtained from the literature.

#### UCM dataset

**Figure 5:**
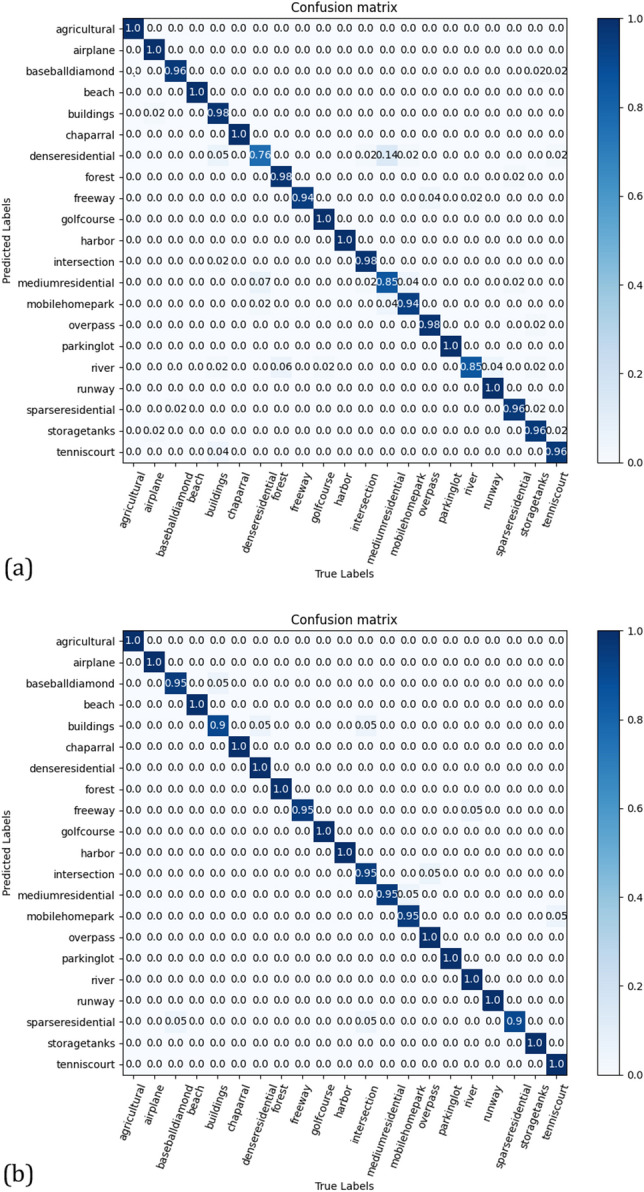
Confusion matrix for the UCM dataset for a training ratio of (**a**) 50% and (**b**) 80% linear assessments.

The experimental results are listed in Table 2. The "–" in Table 2 indicates that the model did not complete the experiment at 50% or 80% of the training rate (the other two datasets are presented in the same form). When the training rate was 50%, APDCNet^47^ used a trainable pooling operation method to improve the training effect, achieving an accuracy of 95.01 ± 0.43%. Our CSAT achieved an accuracy of 95.72 ± 0.23%, which was 0.61% higher than that of APDCNet. When the training rate was 80%, our method achieved an accuracy of 97.86 ± 0.16%, which was 0.81% higher than that of RADC-Net^46^ (which uses residual dense connectivity), 0.76% higher than that of the fine-tuned and pre-trained GoogLeNet^41^ model. ViT-Base^26,30^ and ViT-Large^26,30^ were classified with patch sizes of 16 and 32, respectively, and their accuracies were 95.81% and 96.06%, respectively. Compared to these two methods, our CSAT method achieved an 2.05% and 1.8% improvement, respectively, in terms of accuracy. This not only proves the effectiveness of our method, but also demonstrates that the optimized transformer outperforms certain state-of-the-art (SOTA) methods. The confusion matrix for the UCM test set is shown in Fig. 5.

**Table 2:** Classification results of the UCM dataset.

Method	Training ratio	
	50%	80%
GoogLeNet^41^	92.70 ± 0.60	94.31 ± 0.89
AlexNet^41^	93.98 ± 0.67	95.02 ± 0.81
VGGNet-16^41^	94.14 ± 0.69	95.21 ± 1.20
TEX-Net with VGG^43^	94.22 ± 0.50	95.31 ± 0.69
SPP with AlexNet^44^	94.77 ± 0.46	96.67 ± 0.94
D-CNN with VGGNet-16^45^	–	96.67 ± 0.94
D-CNN with AlexNet^45^	–	97.42 ± 1.79
RADC-Net^46^	94.79 ± 0.42	97.05 ± 0.48
APDCNet^47^	95.01 ± 0.43	97.05 ± 0.43
Fine-tuned GoogLeNet^48^	86.02 ± 0.81	97.10
ViT-Base^26,30^	93.57	95.81
ViT-Large^26,30^	94.00	96.06
T2T-ViT-12^31,49^	95.68 ± 0.61	97.81 ± 0.49
CSAT (ours)	95.72 ± 0.23	97.86 ± 0.16

#### AID dataset

**Figure 6:**
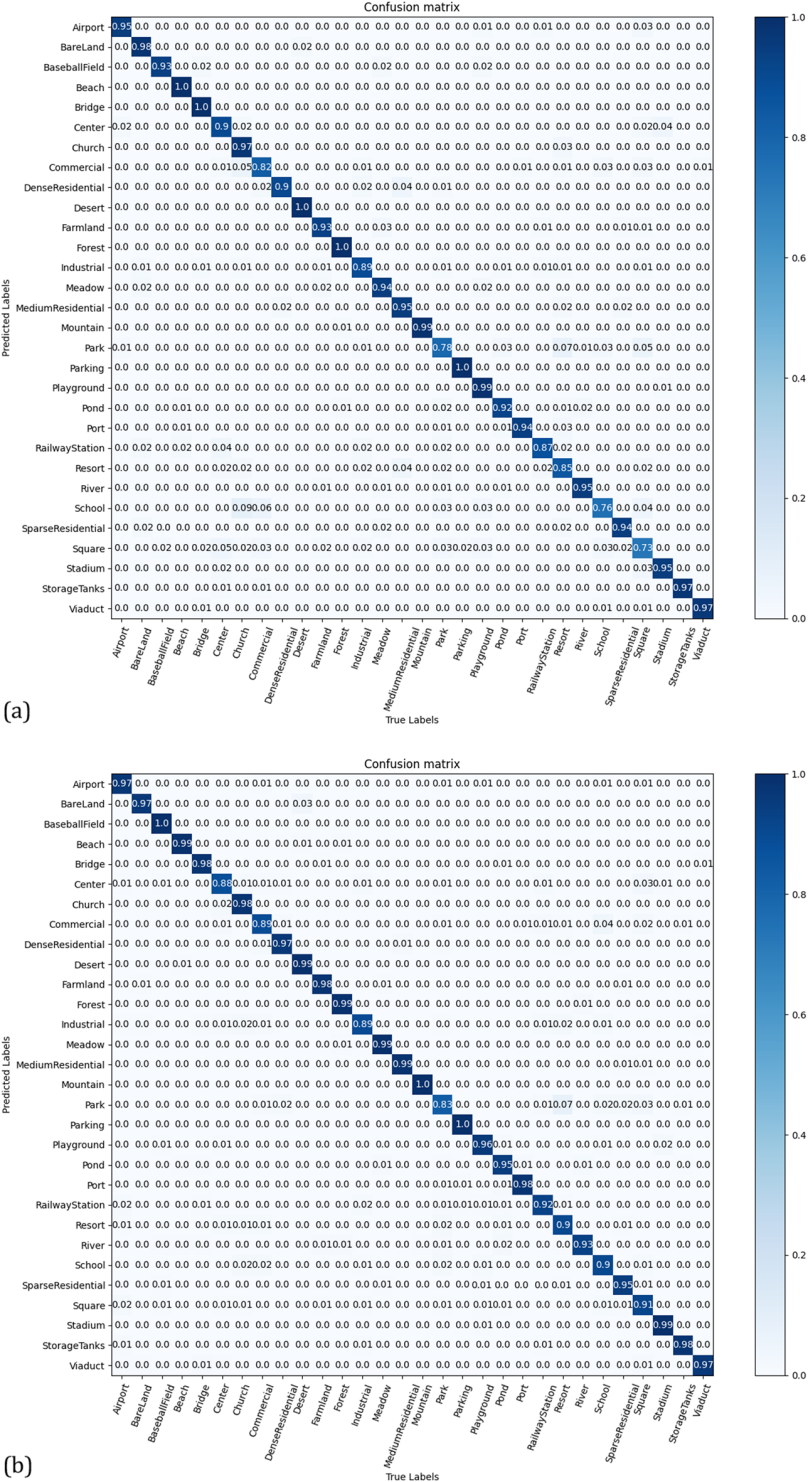
Confusion matrix for the AID dataset for a training ratio of (**a**) 20%, and (**b**) 50% linear assessments.

AID datasets are better than UCM datasets for testing the model performance, as there are more types and numbers of AID datasets than there are UCM datasets. The experimental results are shown in Table 3. At a 20% AID training rate, the accuracy of CSAT was 92.55 ± 0.28%, which was 0.67% higher than that of ViT-Large and 1.39% higher than that of ViT-Base. At a 50% AID training rate, the accuracy of CSAT was 95.44 ± 0.17, which was 0.97% higher than that of D-CNN with AlexNet, 0.13% higher than that of ViT-Large, and 1% higher than that of ViT-Base. The experimental results show that the CSAT performs better on the AID dataset. The confusion matrix for the AID test set is shown in Fig. 6.

**Table 3:** Classification accuracy of AID dataset.

Method	Training ratio	
	20%	50%
GoogLeNet^41^	83.44 ± 0.40	86.39 ± 0.55
AlexNet^41^	86.86 ± 0.47	89.53 ± 0.31
VGGNet-16^41^	86.59 ± 0.29	89.64 ± 0.36
TEX-Net with VGG^43^	87.32 ± 0.37	90.00 ± 0.33
SPP with AlexNet^44^	87.44 ± 0.45	91.45 ± 0.38
D-CNN with AlexNet^45^	85.62 ± 0.10	94.47 ± 0.12
RADC-Net^46^	88.12 ± 0.43	92.35 ± 0.19
MobileNet^50^	88.53 ± 0.17	90.91 ± 0.18
SPP-Net^44^	87.44 ± 0.45	91.45 ± 0.38
Fussion by addition^51^	–	91.87 ± 0.36
ViT-Base^26,30^	91.16	94.44
ViT-Large^26,30^	91.88	95.13
T2T-ViT-12^31,49^	90.09 ± 0.08	93.82 ± 0.55
PiT-S^31,52^	90.51 ± 0.57	94.17 ± 0.36
PVT-Medium^31,53^	92.13 ± 0.45	95.28 ± 0.23
CSAT (ours)	92.55 ± 0.28	95.44 ± 0.17

#### NWPU dataset

**Figure 7:**
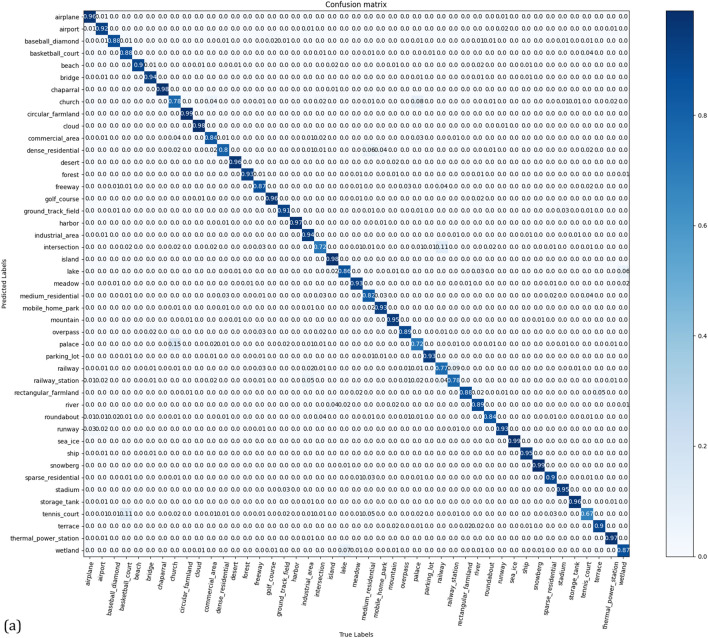

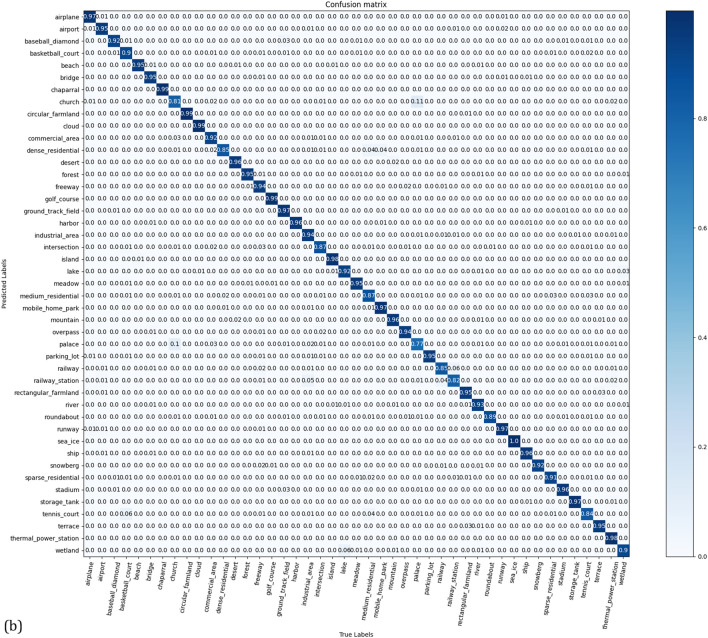
Confusion matrix for the NWPU dataset for a training ratio of (**a**) 10%, and (**b**) 20% linear assessments.

The NWPU dataset has more remote sensing images but is more difficult to train than AID and UCM datasets. The experimental results are listed in Table 4. At an NWPU training rate of 10%, the accuracy of CSAT was 89.70 ± 0.18%, which was 2.11% and 0.54% higher than that of ViT-Base and ViT-Large, respectively. At a training rate of 20%, the accuracy of CSAT was 93.06 ± 0.16%, which was 2.19% and 1.12% higher than that of ViT-Base and ViT-Large, respectively. The experimental results show that the proposed method performs well on the NWPU dataset. The confusion matrix for the NWPU in the test set is shown in Fig. 7.

**Table 4:** Classification accuracy of the NWPU dataset.

Method	Training ratio	
	10%	20%
GoogLeNet^41^	76.19 ± 0.38	78.48 ± 0.26
AlexNet^41^	76.69 ± 0.21	79.85 ± 0.13
VGGNet-16^41^	76.47 ± 0.18	79.79 ± 0.15
SPP with AlexNet^44^	82.13 ± 0.30	84.64 ± 0.23
D-CNN with AlexNet^45^	85.56 ± 0.20	87.24 ± 0.12
RADC-Net^46^	85.72 ± 0.25	87.63 ± 0.28
MobileNet^50^	80.32 ± 0.16	83.26 ± 0.17
SPP-Net^44^	82.13 ± 0.30	84.64 ± 0.23
ViT-Base^26,30^	87.59	90.87
ViT-Large^26,30^	89.16	91.94
T2T-ViT-12^31,49^	84.91 ± 0.30	89.43 ± 0.23
PiT-S^31,52^	85.85 ± 0.18	89.91 ± 0.19
PVT-Medium^31,53^	87.40 ± 0.36	91.36 ± 0.09
CSAT (ours)	89.70 ± 0.18	93.06 ± 0.16

The proposed the CSAT network combines the CSA mechanism to optimize the transformer. As shown in Tables 2, 3 and 4, this method was validated on the UCM, AID, and NWPU datasets and outperformed some existing SOTA models. In addition, the advantages of CSAT compared with some other methods were not significant. For example, TRS^29^ cleverly used ResNet to develop an upgraded version of MHSA, and integrated transformers into CNNs. Based on the basic CNN, CTNet^30^ delicately develops an enhanced version of the CNN-based network. HHTL^31^ carefully designed the patch before it was input to the transformers and subtly fused them after feature extraction. Some methods improved the classification performance by adding operations, e.g., multi-scale, spatial attention, and feature aggregation. However, our CSAT network did not include these advanced skills. In the future, we will attempt to introduce some targeted operations into our CSAT network to improve its performance in terms of HRRS scene classification.

#### Training and testing time and parameters

The training and testing time can directly reflect the efficiency of the model and the time cost of running the model. We use the tqdm package to compare the time required for training and testing the model. As shown in Table 5, the efficiency and time costs of all methods are acceptable. CSAT takes longer to train and test an epoch than ResNet-101 or ResNet-152; however, it outperforms both of them in terms of accuracy. Compared with the SE-Net model, its training time and parameters are higher than those of CSAT. However, the parameters and Flops of the CSAT model are not optimal among models. The reasons for this analysis are as follows. The transformer embedded in the CSAT is the MSA block. It mainly obtains long-term context information from the HRRS scene by measuring the relationship between the HRRS scene patches, which further increases the amount of computation. However, we found that the CSAT model has a slight advantage over the ViT-Base model in terms of time cost, parameter quantity, and Flops, which means that embedding the BAM module into the CSAT model is effective.

**Table5:** Training and testing time and parameter comparison between different models.

Method	UCM (50%)				
	Accuracy	Train (s/epoch)	Test (s/epoch)	Parameters (M)	Flops (G)
ResNet-101^37^	92.47	16.1	6.9	46.0	7.6
ResNet-152^10^	92.95	23.5	9.3	60.0	11.0
SE-Net^54^	95.38	49.7	23.6	146.0	42.0
ViT-Base^26^	93.57	25.9	10.4	86.4	17.5
CSAT (our)	95.72	25.3	10.19	85.99	16.88

### Ablation study for the proposed CSAT

In the ablation study, we explored how the components of the CSAT model affected its performance. To obtain more convincing results, we selected three datasets (i.e., AID, NWPU, and UCM) with different resolutions for the ablation experiments. The training rates of the AID, NWPU, and UCM datasets were chosen to be 50%, 20%, and 80%, respectively.

#### Influence of other attention blocks

We changed the components in the CSAM, which contained the spatial and channel attention modules, as described previously in this paper. We set up the ablation experimental deployment with the channel attention module, and spatial attention module, and replaced CSAM with the channel bottleneck attention module (CBAM). The experimental results are shown in Table 6, in which the results with three sets of experiments are compared. We found that with the channel attention module alone, the classification accuracy of the AID, NWPU, and UCM datasets decreased by 5.08%, 7.26%, and 2.12%, respectively. With the spatial attention module alone, the classification accuracy of the AID, NWPU, and UCM datasets decreased by 6.01%, 8.13%, and 3.09%, respectively. Finally, in the case where the CSAM components were replaced with CBAM, the classification accuracy of the AID, NWPU, and UCM datasets decreased by 3.13%, 4.12%, and 1.07%, respectively. The experimental results show that the CSAM component effectively improves the ViT network performance in the overall model structure.

**Table 6:** Influence of CSAM.

	AID (50%)	NWPU (20%)	UCM (80%)
Channel attention	90.36	85.80	95.74
Spatial attention	89.42	84.93	94.77
CBAM + Transformer	92.31	88.94	96.79
CSAT (ours)	95.44	93.06	97.86

#### Influence of patch size

In this experiment, we changed the size of the 2D flattening patch sequence and set the patch size to 16 and 32. According to the literature^20^, the patch size should be 14, 16, and 32, but due to hardware limitation, the experimental setting was limited to the smallest patch size (i.e., 16). The corresponding results, which indicate that the number of linear embedding sequences input to the encoder module is inversely proportional to patch size^20^, are shown in Table 7. The smaller the patch size, the more pieces are cut; the larger the patch size, the fewer pieces are cut. When the model uses a smaller patch size, the computation is more expensive because the sequence length increases. In this experiment, when the patch size was 32, the accuracy of the AID, NWPU, and UCM datasets decreased by 2.26%, 1.96%, and 2.78%, respectively. This verifies that a large patch size reduces the linear embedding sequence and affects the accuracy.

**Table 7:** Influence of patch size.

Patch size	AID (50%)	NWPU (20%)	UCM (80%)
16	95.44 ± 0.17	93.06 ± 0.16	97.86 ± 0.14
32	93.18 ± 0.23	91.10 ± 0.24	95.08 ± 0.19

### Visual attention map

**Figure 8:**
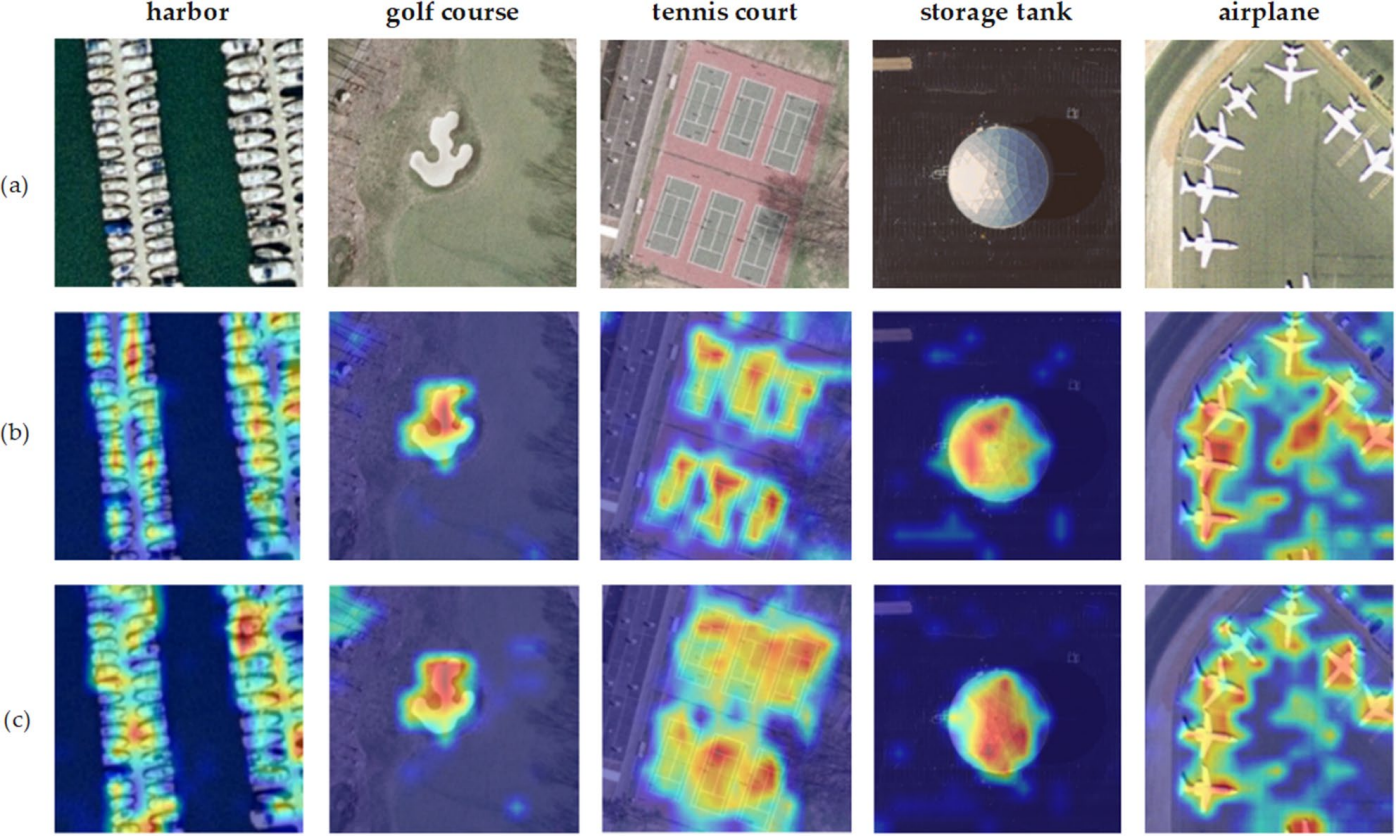
Original scene images and the corresponding attention maps.

To gain insight into the impact of the CSAM components on the proposed model performance, we provide some visualization examples, which are shown in Fig. 8. Figure 8a,b show the original scene image, the image without CSAM components, and the image with CSAM components, respectively. The area highlighted in the feature map indicates a greater significance assigned to the classification of that area. Five scenes were selected, namely, harbor, golf course, tennis court, storage tank, and airplane. These results suggest that the network structure with CSAM components extracts the discriminative information of these scenes more accurately. The main target in each scene was more accurately captured, which greatly promoted scene classification and reflected the positive effect of CSAM components on the overall network structure.

## Conclusion

In this paper, a CSAT network combined with a channel-spatial attention (CSA) mechanism to optimize the ViT network is proposed for HRRS image scene classification. The proposed network first processes the HRRS image into a linear embedding sequence, uses the CSA module to extract fine-grained image features, and then enters the transformer encoder. There, the core MSA module resolves the long-range dependencies between HRRS images while discarding convolutional operations to avoid information loss caused by the irregular processing of typical convolutional kernels during classification. Overall, the proposed CSAT network combines the CSAM module, MSA, linear mapping, regularization, activation function, and other operations and utilizes the residual structure to form the encoder blocks. To improve the performance of the CSAT network, multiple encoder blocks were stacked to form the main model structure. We conducted two sets of experiments using three public datasets to validate the effectiveness of the proposed model. The first set of experiments defined the training ratio of the dataset, and the proposed model was then compared with a selection of existing SOTA classification methods, including ViT-Base, ViT-Large, RADC-NET, and GoogLeNet. The experimental results show that the proposed CSAT model outperforms the SOTA methods. One of the components of the CSAT network is the CSA component. In this component, we selected the BAM module, which divided the attention process into two independent parts (i.e., the channel and spatial attention modules), and fused the attention weights of these two levels in parallel. The BAM module is a simple and effective attention module, which could be integrated into existing network architectures as a plug-and-play module with higher flexibility^16^. In the second set of experiments, we analyzed the effect of the CSA component on the model performance by removing the spatial and channel attention modules for comparison, as well as replacing the CSA component with the CBAM component. The experimental results indicated that CSA was more beneficial to the performance of the CSAT model. By changing the hyperparameter patch size, the experimental results illustrated that a patch size of 36 decreases the number of linearly embedded sequences and affects the classification accuracy.

In conclusion, the classification results of the CSAT network for the UCM, AID, and NWPU datasets significantly outperformed a selection of existing SOTA methods, thereby illustrating the effectiveness of our proposed network. However, our proposed method still has some limitations: the method validation is based on public datasets and lacks real data application. In future work, we will explore the application of real remote sensing images based on deep learning methods^55^. For example, the proposed CSAT network will be applied to crop identification, a field where deep learning is widely used. According to a literature survey^56^, deep learning is rarely used for particular special crops, such as medicinal plants. Due to the lack of data sets for such crops and their low coverage, the relative fragmentation of acreage compared to conventional crops such as large maize and wheat, introduces certain difficulties into the research. Therefore, capturing the characteristic information of the limited medicinal plant data through the proposed CSAT network will be part of our future work. Such research will play an important role in achieving the sustainable use of medicinal plant resources, the coordinated development of economic and social resources, and the conservation of the ecological balance.

## Data Availability

The three public remote sensing scene image datasets used in this study are available in the following sites: (1) UC-Merced:http://weegee.vision.ucmerced.edu/datasets/landuse.html; (2) AID: http://captain-whu.github.io/AID/; (3) NWPU-RESISC45: http://www.escience.cn/people/JunweiHan/NWPU-RESISC45.html. The author commit to sharing the raw data and materials upon acceptance of the Stage 2 manuscript.

## Code availability

The datasets generated and analyzed during the current study are available in the gjx2017/csam repository, https://github.com/gjx2017/csam. The authors commit to sharing all codes upon acceptance of the Stage 2 manuscript.

## References

[1] Wang Q (2021). Ship detection based on fused features and rebuilt YOLOv3 networks in optical remote-sensing images. Int J. Remote Sens..

[2] Liu H (2019). DE-Net: Deep encoding network for building extraction from high-resolution remote sensing imagery. Remote Sens..

[3] Ren Y, Yu Y, Guan H (2020). DA-CapsUNet: A dual-attention capsule U-net for road extraction from remote sensing imagery. Remote Sens..

[4] Huang X, Chen H, Gong J (2018). Angular difference feature extraction for urban scene classification using ZY-3 multi-angle high-resolution satellite imagery. ISPRS J. Photogramm. Remote Sens..

[5] Han W (2021). Methods for small, weak object detection in optical high-resolution remote sensing images: A survey of advances and challenges. IEEE Geosci. Remote Sens..

[6] Li, K. *et al*. Object detection in optical remote sensing images: A survey and a new benchmark, *arXiv2019*, arXiv:1909.00133v1 (2019).

[7] Alsharrah SA (2016). Use of shadow for enhancing mapping of perennial desert plants from high-spatial resolution multispectral and panchromatic satellite imagery. J. Appl Remote Sens..

[8] Ghazouani F, Farah IR, Solaiman BA (2019). Multi-level semantic scene interpretation strategy for change interpretation in remote sensing imagery. IEEE Trans. Geosci. Remote Sens..

[9] Cheng, G. *et al*. Remote sensing image scene classification meets deep learning: Challenges, methods, benchmarks, and opportunities. *IEEE J. Sel. Top Appl Earth Obs. Remote Sens*. **13**, 3735–3756 (2020).

[10] Zhang X, Du S (2015). A Linear Dirichlet Mixture Model for decomposing scenes: Application to analyzing urban functional zonings. Remote Sens. Environ..

[11] Gong C, Han J, Lu X (2017). Remote sensing image scene classification: Benchmark and state of the art. Proc. IEEE.

[12] Ma L, Liu Y (2019). Deep learning in remote sensing applications: A meta-analysis and review. ISPRS J. Photogramm. Remote Sens..

[13] Wan H (2021). Lightweight channel attention and multiscale feature fusion discrimination for remote sensing scene classification. IEEE Access.

[14] Mei, S. *et al*. Remote sensing scene classification using sparse representation-based framework with deep feature fusion. *IEEE J. Sel. Top Appl Earth Obs. Remote Sens.* **14**, 5867–5878 (2021).

[15] Yuan Y, Fang J, Lu X, Feng Y (2019). Remote sensing image scene classification using rearranged local features. IEEE Trans. Geosci. Remote Sens.

[16] Park, J., Woo, S., Lee, J.Y. & Kweon, I.S. BAM: Bottleneck Attention Module, *arXiv2018*, arXiv:1807.06514v2 (2018).

[17] Woo, S., Park, J., Lee, J.Y. & Kweon, I.S. CBAM: Convolutional Block Attention Module, *arXiv 2018*, arXiv:1807.06521v1 (2018).

[18] Yu, D.*et al*. Hierarchical attention and bilinear fusion for remote sensing image scene classification. *IEEE J. Sel. Top Appl Earth Obs. Remote Sens*. **13**, 6372–6383 (2020).

[19] Tong, W.*et al*. Channel-attention-based densenet network for remote sensing image scene classification. *IEEE J. Sel. Top Appl. Earth Obs. Remote Sens*. **13**, 4121–4132 (2020).

[20] Ma, W. *et al.* A multi-scale progressive collaborative attention network for remote sensing fusion classification. *IEEE Trans. Neural Netw. Learn Syst.* 1–15 (2021). 10.1109/TNNLS.2021.3121490 34714755

[21] Zhu H (2021). A spatial-channel progressive fusion ResNet for remote sensing classification. Inf. Fusion.

[22] Zhu H (2020). A dual–branch attention fusion deep network for multiresolution remote–sensing image classification. Inf. Fusion.

[23] Ma W (2020). A spatial-channel collaborative attention network for enhancement of multiresolution classification. Remote Sens.

[24] Li, F.*et al*. An Augmentation attention mechanism for high-spatial-resolution remote sensing image scene classification. *IEEE J. Sel. Top Appl Earth Obs. Remote Sens*, **13**, 3862–3878 (2020).

[25] Guo Y (2019). Global-local attention network for aerial scene classification. IEEE Access.

[26] Dosovitskiy, A. *et al*. An image is worth 16x16 words: Transformers for image recognition at scale, *arXiv2020*, arXiv:2010.11929 (2020).

[27] Yi, T., Dehghani, M., Bahri, D. & Metzler, D. Efficient transformers: A Survey, *arXiv2020*, arXiv:2009.06732v2 (2020).

[28] Bazi Y (2021). Vision transformers for remote sensing image classification. Remote Sens..

[29] Deng P, Xu K, Huang H (2021). When CNNs meet vision transformer: A joint frame work for remote sensing scene classification. IEEE Geosci. Remote Sens. Lett..

[30] Li J, Zhang J, Zhao H (2021). TRS: Transformers for remote sensing scene classification. Remote Sens..

[31] Ma, J*.et al*. Homo–heterogenous transformer learning framework for RS scene classification. *IEEE J. Sel. Top Appl Earth Obs. Remote Sens*. **15**, 2223–2239 (2022).

[32] Vaswani, A. *et al*. Attention Is All You Need, *arXiv 2017*, arXiv:1706.03762v5 (2017).

[33] d’Ascoli, S., Touvron, H., & Leavitt, M. L. *et al*. Convit: Improving vision transformers with soft convolutional inductive biases, *arXiv2021*, arXiv:2103.10697v2 (2021).

[34] Cordonnier, J.B., Loukas, A., & Jaggi, M. On the relationship between self-attention and convolutional layers, *arXiv:2019*, arXiv:1911.03584(2019).

[35] Bello, I. *et al*. Attention augmented convolutional networks, *Proceedings of the IEEE/CVF international conference on computer vision (ICCV)*, Soul, Korea (South), 27 Oct.-2 Nov. pp. 3286–3295 (2019).

[36] Ramachandran, P., Parmar, N., Vaswani, A. *et al*. Stand-alone self-attention in vision models, *arXiv:2019*, arXiv:1906.05909v1(2019).

[37] He, K.M., Zhang, X., Ren, S. & Sun, J. Deep Residual Learning for Image Recognition, *IEEE Conference on Computer Vision and Pattern Recognition (CVPR)*, Las Vegas, NV, USA, 27–30 June pp. 770–778 (2016).

[38] Ba, J.L., Kiros, J.R. & Hinton, G.E. Layer normalization, *arXiv2016*, arXiv:1607.06450 (2016).

[39] Hendrycks, D. & Gimpel, K. Gaussian Error Linear Units (GELUs), *arXiv2016*, arXiv:1606.08415v4 (2016).

[40] Yang Y, Newsam S (2013). Geographic image retrieval using local invariant features. IEEE Trans. Geosci. Remote Sens..

[41] Xia G (2017). AID: A benchmark data set for performance evaluation of aerial scene classification. IEEE Trans. Geosci. Remote Sens..

[42] Simonyan, K. & Zisserman, A. Very deep convolutional networks for large-scale image recognition. *arXiv2014*, arXiv:1409.1556(2014).

[43] Anwer RM (2018). Binary patterns encoded convolutional neural networks for texture recognition and remote sensing scene classification. ISPRS J. Photogramm. Remote Sens..

[44] Han, X., Zhong, Y., Cao, L. & Zhang, L. Pre-Trained AlexNet Architecture with pyramid pooling and supervision for high spatial resolution remote sensing image scene classification. *Remote Sens*. 848 (2017).

[45] Cheng G (2018). When deep learning meets metric learning: Remote sensing image scene classification via learning discriminative CNNs. IEEE Trans. Geosci. Remote Sens..

[46] Bi Q (2020). RADC-Net: A residual attention based convolution network for aerial scene classification. Neurocomputing.

[47] Bi Q (2019). APDC-Net: Attention pooling-based convolutional network for aerial scene classification. IEEE Geosci. Remote Sens. Lett..

[48] Gong C (2017). Remote sensing image scene classification using bag of convolutional features. IEEE Geosci. Remote Sens. Lett..

[49] Yuan, L. *et al*. Tokens-to-token vit: Training vision transformers from scratch on imagenet, *Proceedings of the IEEE/CVF International Conference on Computer Vision (ICCV)*.11–17 Oct., pp. 558–567 (2021).

[50] Pan H (2020). A new image recognition and classification method combining transfer learning algorithm and MobileNet model for welding defects. IEEE Access.

[51] Chaib S, Liu H, Gu Y (2017). Deep feature fusion for VHR remote sensing scene classification. IEEE Trans. Geosci. Remote Sens..

[52] Heo, B. *et al*. Rethinking spatial dimensions of vision transformers, *Proceedings of the IEEE/CVF International Conference on Computer Vision (ICCV)*.11–17 Oct., pp. 11936–11945 (2021).

[53] Wang, W. *et al*. Pyramid vision transformer: A versatile backbone for dense prediction without convolutions, *Proceedings of the IEEE/CVF International Conference on Computer Vision (ICCV)*. 11–17 Oct., pp. 568–578 (2021).

[54] Hu, J. *et al*. Squeeze-and-excitation networks. *In Proceedings of the IEEE Conference on Computer Vision and Pattern Recognition (CVPR)*, Salt Lake City, UT, USA, 18–22 Jun., pp. 7132–7141(2018).

[55] Ma L (2019). Deep learning in remote sensing applications: A meta-analysis and review. ISPRS J. Photogramm. Remote Sens..

[56] Guo J (2021). Application of remote sensing technology in medicinal plant resources. Chi. J. Chin. Mater. Med..

